# Case Report: Left Vocal Cord Palsy and Hoarseness—A Case of Mycotic Arch Aneurysm With Infective Endocarditis

**DOI:** 10.1155/crot/8439769

**Published:** 2026-05-26

**Authors:** Shrotriya Sen, Daniel Jason Lin

**Affiliations:** ^1^ ENT Department, Darlington Memorial Hospital, County Durham and Darlington NHS Foundation Trust, Darlington, UK, cddft.nhs.uk

## Abstract

We present contrast‐enhanced CT images of the neck and chest from a gentleman in his 70s who presented with fever, malaise, and left knee pain. He was diagnosed with septic arthritis and *Staphylococcus aureus* bacteremia. During admission, he developed new‐onset hoarseness. Flexible laryngoscopy confirmed left vocal cord palsy. CT neck and thorax with contrast revealed a saccular aneurysm of the aortic arch (Standring, 2020; Netter, 2022; Paquette et al., 2012). Urgent surgical repair revealed an aortic abscess with rupture into the pericardial cavity. He was treated for infective endocarditis with intravenous flucloxacillin. Postoperatively, he developed a superficial sternotomy wound infection due to *Candida albicans*, successfully managed with fluconazole and negative‐pressure wound therapy. Follow‐up CT angiogram confirmed stability of the repaired aortic arch (Standring, 2020; Netter, 2022; Paquette et al., 2012). Ongoing dysphonia raised concern for persistent left recurrent laryngeal nerve palsy, prompting SALT referral for rehabilitation.

## 1. Introduction

Vocal fold paralysis reflects impaired vocal fold mobility, the most common cause being the dysfunction of the nerve supplying the vocal cord. The recurrent laryngeal nerve (RLN) is a branch of the vagus nerve that normally controls movement of the vocal cords by controlling laryngeal muscle movements [1]. Unilateral vocal cord palsy is more common and presents as dysphonia (hoarseness of voice), vocal fatigue, or aspiration, whereas bilateral palsy can be life‐threatening and presents with emergency airway compromise (stridor) [2]. The etiology of vocal cord palsy is broad and includes iatrogenic injury during thoracic or neck surgery, malignancies (especially of the lung, thyroid, or esophagus), neurologic disorders, viral infections, and idiopathic causes [2, 3]. In the context of thoracic surgery, particularly procedures involving the aortic arch or mediastinum, the RLN is vulnerable to direct trauma from traction or compression due to its long, looping anatomical course, particularly on the left side [3–5].

Cardiovocal (Ortner) syndrome refers to hoarseness caused by left RLN palsy due to cardiovascular or mediastinal vascular pathology, which, by mass effect, disturbs the long looping course of the left RLN in the mediastinum [6]. Examples include aortic aneurysm, severe dilatation of the left atrium, or pulmonary artery hypertension with enlarged pulmonary artery.

This case illustrates a rare presentation of a mycotic aortic arch aneurysm manifesting initially as generalized weakness and hoarseness of voice, with subsequent complications including pericardial rupture, sternal wound infection with fungal colonization, and vocal cord weakness. The multidisciplinary approach, spanning cardiothoracic surgery, infectious disease, wound care, and ENT rehabilitation, was critical in the patient’s recovery.

## 2. Clinical Summary

A gentleman in his mid‐70s presented to the emergency department on December 5, 2024, with general malaise, pyrexia, and impaired mobility, particularly due to pain and reduced range of motion in the left knee. Initial clinical suspicion pointed toward septic arthritis, later confirmed by positive blood cultures for *Staphylococcus aureus*. He was reviewed by the orthopedic team and initiated on intravenous antibiotics.

During his inpatient course, the patient developed a new‐onset hoarseness. ENT review with flexible laryngoscopy revealed an isolated left vocal cord palsy. A CT scan of the thorax revealed a saccular aneurysm of the aortic arch [3–5]. Given the potential for rupture, he was transferred to a tertiary cardiothoracic center on December 12, 2024. He underwent surgical repair of the aneurysm on December 16, during which an aortic arch abscess was discovered with extension into the pericardial cavity. Intraoperative findings and persistently positive blood cultures prompted initiation of a 6‐week course of intravenous flucloxacillin, with a working diagnosis of infective endocarditis. Because *Staphylococcus aureus* bacteremia carries a clinically important risk of occult infective endocarditis, the diagnostic framework for IE was assessed using Duke‐based criteria [7]. In the modified Duke approach, *S. aureus* bacteremia constitutes a major criterion; the presence of an infected (“mycotic”) thoracic aneurysm constitutes a vascular phenomenon as a minor criterion, and fever constitutes a minor criterion. Based on the information available at the time, these findings fulfilled the criteria for possible infective endocarditis. While definitive IE would require documented endocardial involvement (e.g., vegetation or perivalvular abscess) on transthoracic/transoesophageal echocardiography or equivalent major imaging/surgical criteria, given the severity of invasive *S. aureus* infection and the presence of a high‐risk vascular complication requiring urgent source control, prolonged intravenous anti‐staphylococcal therapy was administered in parallel with operative management.

Postoperatively, the patient developed purulent discharge from the upper aspect of the sternotomy site. Bedside drainage of a superficial collection was performed, and negative‐pressure wound therapy (PICO dressing) was applied. Swab cultures from the wound later yielded *Candida albicans*, for which fluconazole therapy was commenced as per microbiological guidance [8].

He was transferred back to the district general hospital on January 1, 2025, where wound care continued with PICO dressing. The dressing was changed on January 1, 2025. The patient completed both his courses of IV flucloxacillin and oral fluconazole. Follow‐up CT angiography demonstrated a stable postoperative aortic arch repair without progression of the aneurysm.

Given the patient’s persistent dysphonia and left RLN involvement, the ENT team recommended referral to the speech and language therapy (SALT) team for rehabilitative voice therapy [9].

## 3. Conclusion

This case highlights a rare but high‐stakes cause of unilateral vocal fold paralysis: an infected aortic arch aneurysm in the setting of *Staphylococcus aureus* bacteremia [10], discovered after new‐onset hoarseness that led to laryngoscopic confirmation of isolated left vocal fold paralysis and subsequent thoracic imaging. The patient required urgent surgical intervention, which identified an aortic arch abscess with pericardial extension, and he subsequently completed prolonged antimicrobial therapy for presumed infective endocarditis. Postoperative superficial sternal wound infection due to *Candida albicans* was successfully managed with targeted antifungal therapy and negative‐pressure wound therapy, and follow‐up CTA demonstrated stable aortic repair.

While cardiovocal (Ortner) syndrome is a recognized mechanism for hoarseness, it remains an uncommon etiology of RLN palsy [6] and is frequently overlooked in otolaryngology‐first presentations. This case is notable because (1) hoarseness emerged during bacteremia and preceded diagnosis of a thoracic vascular emergency; (2) intraoperative findings established an infected aortic arch process with pericardial extension, an anatomic pattern associated with potentially catastrophic deterioration; and (3) the patient’s course illustrates both the necessity of multidisciplinary source control and the practical downstream need for voice rehabilitation when dysphonia persists. The left RLN’s intrathoracic route places it at risk of compression or stretch where it traverses the aortopulmonary window beneath the aortic arch [3–5].

Enlargement of thoracic vascular structures, including aneurysms of the arch or proximal descending aorta, can therefore produce hoarseness via left RLN palsy [3, 6]. In our patient, the mechanism is plausibly multifactorial: mechanical compression/stretch from the aneurysm sac and local inflammatory involvement from an infected aortic wall/abscess. This is biologically consistent with radiologic–pathologic descriptions of infected (“mycotic”) aneurysms in which infectious arteritis destroys the arterial wall, leading to rapid pseudoaneurysm formation, periaortic inflammatory tissue, and is associated with a risk of rupture [11, 12].

The literature repeatedly shows that patients with hoarseness and unilateral vocal fold paralysis often present first to ENT services, and in the absence of chest pain or dysphagia, aortic imaging may be delayed. In the Zhang case series, three of four patients presented initially with hoarseness, attended otolaryngology first, and were later diagnosed with thoracic aortic aneurysm by CT after delays ranging from 1 to 24 months, highlighting the importance of early mediastinal evaluation [3, 6] when laryngoscopy confirms left vocal cord paralysis [13]. Similar patterns appear in other reports where hoarseness is prolonged and isolated.

From a radiology perspective, CT angiography is a principal diagnostic tool for suspected infected aneurysm because it defines aneurysm morphology, detects periaortic inflammatory change, and supports urgent operative planning [3, 11]. In ENT practice, this case supports a pragmatic rule: unexplained unilateral (especially left‐sided) vocal fold paralysis warrants imaging along the RLN course, including the chest, to exclude mediastinal malignancy and thoracic aortic pathology.

Management of infected thoracic/aortic arch aneurysms generally requires (a) source control (open repair, endovascular repair, or hybrid strategies depending on anatomy and sepsis stability) and (b) prolonged targeted antimicrobials [11, 12]. Modern reviews emphasize that delayed recognition is associated with worse outcomes and that infected aneurysms can rapidly enlarge or rupture. Systematic reviews comparing open and endovascular approaches suggest evolving roles for endovascular repair, including bridge‐to‐surgery strategies in unstable patients, while also recognizing the ongoing risk of persistent/recurrent infection and the difficulty of randomized evaluation in this rare condition [12].

In the cardiovocal syndrome reports, definitive treatment of the aneurysm can improve hoarseness in some patients, but recovery is variable. In Zhang et al.’s series, one patient had complete resolution within 6 months after TEVAR and two improved; one had no improvement [13]. Garrido et al.’s case showed reduction of hoarseness after open repair in a giant aneurysm [14], whereas Mesquita et al.’s case reported persistent hoarseness despite aneurysm correction, attributed to prolonged stretching and axonal injury [15]. These observations suggest that time‐to‐diagnosis and duration of RLN compression/inflammation may be important determinants of voice recovery, supporting rapid imaging and treatment where feasible.

Clinically, this case supports an algorithmic approach: after confirming unilateral vocal fold paralysis, clinicians should evaluate along the RLN pathway and maintain suspicion for thoracic aortic pathology, particularly when systemic infection is present, because early CTA and surgical consultation can be life‐saving. Future research priorities include (a) multicenter registries of infected thoracic/aortic arch aneurysms presenting with ENT symptoms to characterize diagnostic latency and outcomes; (b) comparative effectiveness studies of open versus endovascular strategies specifically in infected arch aneurysms; and (c) prognostic studies on voice recovery after aneurysm repair, incorporating duration of RLN dysfunction and imaging markers of nerve compression/inflammation.

## Funding

No funding was received for this study.

## Conflicts of Interest

The authors declare no conflicts of interest.

## Supporting Information

Additional supporting information can be found online in the Supporting Information section.

## Supporting information


**Supporting Information** Supporting file (IMAGESPub.docx) contains Figures S1–S4, including contrast‐enhanced CT images of the neck and thorax demonstrating the aortic arch aneurysm and imaging features of left vocal fold paralysis.

## Data Availability

The data that support the findings of this study are available from the corresponding author upon reasonable request.

## References

[1] Sarangi P. K. , Hui P. , Sagar H. S. , Kisku D. K. , and Mohanty J. , Combined Left Recurrent Laryngeal Nerve and Phrenic Nerve Palsy: A Rare Presentation of Thoracic Aortic Aneurysm, Journal of Clinical and Diagnostic Research. (2017) 11, no. 5, TD01–TD02, 10.7860/JCDR/2017/25035.9765, 2-s2.0-85018445533. PMC5483778 28658876

[2] Bhatta S. , Gandhi S. , Ghanpur A. D. , and Ganesuni D. , Etiology and Presenting Features of Vocal Cord Paralysis: Changing Trends over the Last Two Decades, Egyptian Journal of Otolaryngology. (2022) 38, no. 1, 10.1186/s43163-022-00322-x.

[3] Paquette C. M. , Manos D. C. , and Psooy B. J. , Unilateral Vocal Cord Paralysis: A Review of CT Findings, Mediastinal Causes, and the Course of the Recurrent Laryngeal Nerves, Radiographics. (2012) 32, no. 3, 721–740, 10.1148/rg.323115129, 2-s2.0-84861179291. 22582356

[4] Standring S. , Gray’s Anatomy: The Anatomical Basis of Clinical Practice, 2020, 42nd edition, Elsevier.

[5] Netter F. H. , Atlas of Human Anatomy, 2022, 8th edition, Elsevier.

[6] Verma S. , Talwar A. , Talwar A. , Khan S. , Krishnasastry K. V. , and Talwar A. , Ortner’s Syndrome: A Systematic Review of Presentation, Diagnosis and Management, Intractable & Rare Diseases Research. (2023) 12, no. 3, 141–147, 10.5582/irdr.2023.01047. 37662622 PMC10468413

[7] Delgado V. , Ajmone Marsan N. , de Waha S. et al., 2023 ESC Guidelines for the Management of Endocarditis, European Heart Journal. (2023) 44, no. 39, 3948–4042, 10.1093/eurheartj/ehad193. 37622656

[8] Pappas P. G. , Kauffman C. A. , Andes D. R. et al., Clinical Practice Guideline for the Management of Candidiasis: 2016 Update by the Infectious Diseases Society of America, Clinical Infectious Diseases. (2016) 62, no. 4, e1–e50, 10.1093/cid/civ933, 2-s2.0-84959876270. 26679628 PMC4725385

[9] Alegría R. , Vaz Freitas S. , and Manso M. C. , Efficacy of Speech-Language Therapy Intervention in Unilateral Vocal Fold Paralysis: A Systematic Review and Meta-Analysis, Logopedics Phoniatrics Vocology. (2021) 46, no. 2, 86–98, 10.1080/14015439.2020.1762730. 32406287

[10] Tong S. Y. , Davis J. S. , Eichenberger E. , Holland T. L. , and Fowler V. G.Jr., *Staphylococcus aureus* Infections: Epidemiology, Pathophysiology, Clinical Manifestations, and Management, Clinical Microbiology Reviews. (2015) 28, no. 3, 603–661, 10.1128/cmr.00134-14, 2-s2.0-84930239306. 26016486 PMC4451395

[11] Isselbacher E. M. , Preventza O. , Black J. H.3rd et al., 2022 ACC/AHA Guideline for the Diagnosis and Management of Aortic Disease, Circulation. (2022) 146, no. 24, e334–e382. 36322642 10.1161/CIR.0000000000001106 PMC9876736

[12] Li H. L. , Kwan K. J. S. , Chan Y. C. , and Cheng S. W. , Contemporary Outcomes of Endovascular and Open Surgical Repair for Mycotic Aortic Aneurysms: A Systematic Review, Annals of Vascular Surgery. (2024) 100, 172–183, 10.1016/j.avsg.2023.08.039. 37898457

[13] Zhang Z. , Feng H. , Chen X. , and Li W. , Ortner’s Syndrome Secondary to Thoracic Aortic Aneurysm: A Case Series, Journal of Cardiothoracic Surgery. (2022) 17, no. 1, 10.1186/s13019-022-02023-1. PMC9583463 36266693

[14] Garrido J. M. , Esteban M. , Lara J. , Rodriguez-Vazquez J. F. , Verdugo-Lopez S. , and Lopez-Checa S. , Giant Aortic Arch Aneurysm and Cardio-Vocal Syndrome: Still an Open-Surgery Indication, Cardiology Research. (2011) 2, no. 6, 304–306, 10.4021/cr101w. 28352401 PMC5358261

[15] Mesquita A. , Maximiano P. , Susa M. , and Cruz R. , Cardiovocal Syndrome- An Aortic Arch Aneurysm as a Rare Cause of Vocal Cord Paralysis, Portuguese Journal of Cardiac Thoracic and Vascular Surgery. (2022) 29, no. 3. 10.48729/pjctvs.268 36197825

