# Supplementary material for: Case Report: Left Vocal Cord Palsy and Hoarseness—A Case of Mycotic Arch Aneurysm With Infective Endocarditis
Source: Case Rep Otolaryngol. 2026 May 26;2026:8439769. doi: 10.1155/crot/8439769 (PMC13203783; doi:10.1155/crot/8439769)
Supplement: Supplementary file 1 — Supporting Information Supporting file (IMAGESPub.docx) contains Figures S1–S4, including contrast‐enhanced CT images of the neck and thorax demonstrating the aortic arch aneurysm and imaging features of left vocal fold paralysis. [file CROT-2026-8439769-s001.docx]

**CASE REPORT: Left Vocal Cord Palsy and Hoarseness: A Case of Mycotic Arch Aneurysm with Infective Endocarditis**

***Dr.Shrotriya Sen^1^ , Mr. Daniel Jason Lin^1^**

**¹ENT Department, Darlington Memorial Hospital, County Durham and Darlington NHS Foundation Trust, UK**

**Corresponding Author: Dr. Shrotriya Sen, Email:** [**shrotriya.sen@nhs.net**](mailto:shrotriya.sen@nhs.net)

**Fig S1.**


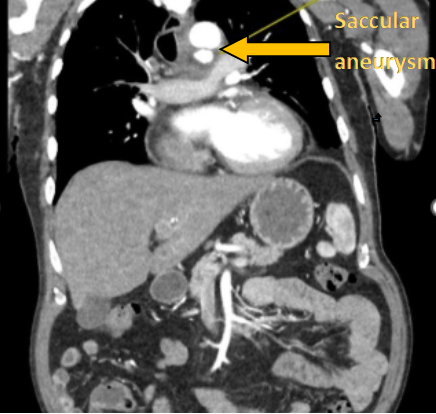


**Figure S1. Coronal contrast-enhanced CT of the neck and chest showing a focal saccular aneurysm of the aortic arch (arrow).**

Contrast-enhanced CT angiography (coronal Multi Planar Reformation, MPR) demonstrates a focal saccular outpouching of the aortic arch (arrow/marker), consistent with a saccular aneurysm/pseudoaneurysm. In the setting of S. aureus bacteraemia and operative aortic abscess, imaging supports infectious aortitis / infected thoracic aneurysm with high rupture risk and a plausible mechanism for hoarseness via left RLN compression.

**Fig S2**


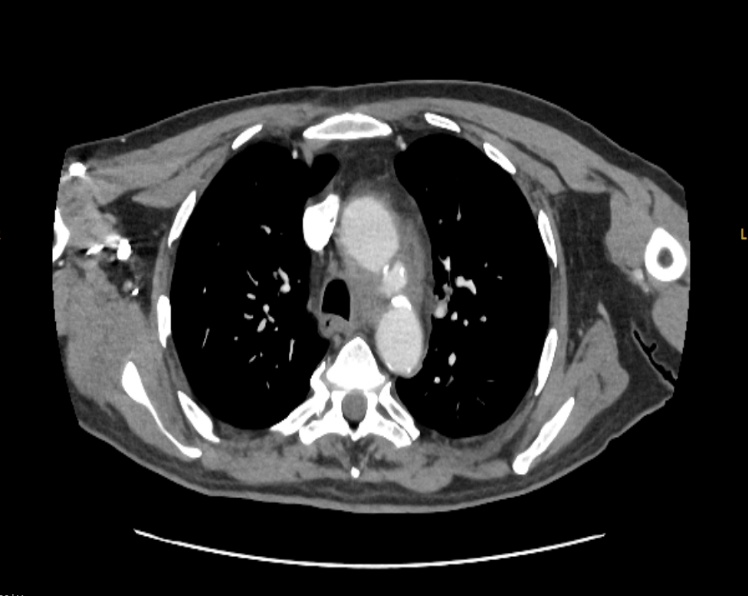


| **Figure S2. Axial contrast-enhanced CT demonstrating the aortic arch aneurysm.**  Contrast-enhanced CT angiography (axial) at the aortic arch shows an eccentric/saccular aneurysmal component. Also showing signs of periaortic inflammation (periaortic soft tissue stranding, rim-enhancing periaortic tissue, gas). Differential for a focal saccular arch lesion includes penetrating atherosclerotic ulcer, traumatic pseudoaneurysm, and infected aneurysm; bacteraemia and operative abscess strongly favour infection |
| --- |

**Fig S3.**


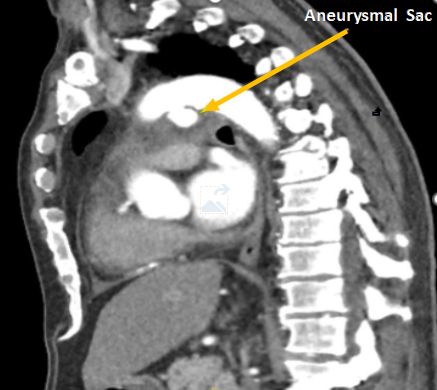


**Figure S3. Sagittal reconstruction highlighting the aortic arch aneurysm (annotated).**

Contrast-enhanced CT angiography (sagittal MPR) depicts the aneurysmal sac arising from the aortic arch, demonstrating its origin/neck orientation for surgical planning. This anatomic pattern can produce cardiovocal (Ortner) syndrome via mass effect along the intrathoracic course of the left recurrent laryngeal nerve.

**Fig S4.**

**
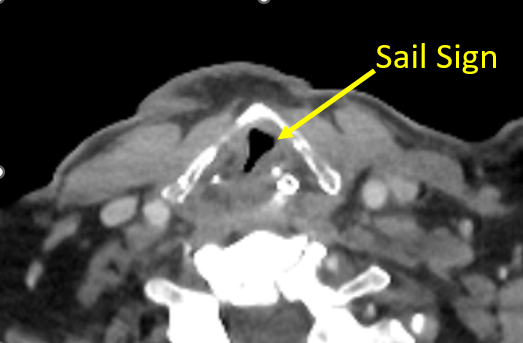
**

**Figure S4. Axial contrast-enhanced CT at the glottic level demonstrating imaging features consistent with left vocal fold paralysis, including the “sail sign” (dilation of the left laryngeal ventricle) (annotated).**

CT neck (axial, soft-tissue window) at the glottic level shows indirect signs of left vocal fold paralysis, including dilation of the left laryngeal ventricle (“sail sign”). Also shows medial displacement of the posterior left vocal fold margin and/or ipsilateral pyriform sinus dilation. These CT findings corroborate laryngoscopic left vocal fold immobility and support a mediastinal aetiology when paired with arch pathology.
